# Conclusions: Towards a sociology of pandemics and beyond

**DOI:** 10.1177/00113921211023518

**Published:** 2021-07

**Authors:** Jens O Zinn

**Affiliations:** University of Melbourne, Australia

**Keywords:** COVID-19, material semiotics, new normal, pandemic, risk, risk society, social change, social learning, social media

## Abstract

This conclusion revisits the COVID-19 pandemic from the broader perspective of a changing global world. It raises questions regarding the opportunities for global learning under conditions of global divisions and competition and includes learning from the Other, governing within a changing public sphere, and challenging national cultural practices. Moreover, it exemplifies how the society–nature–technology nexus has become crucial for understanding and reconstructing the dynamics of the coronavirus crisis such as the assemblages of geographical conditions, technological means and the governing of ignorance, the occurrence of hotspots as well as living under lockdown conditions. It finishes with some preliminary suggestions how reoccurring pandemics might contribute to long-term changes in human attitudes and behaviour towards the environment and a technologically shaped lifeworld.

## Introduction

Epidemics are reoccurring events that significantly affect societies or sometimes even threaten their existence. Considering repeating global pandemics, sociologists of health and illness have suggested developing a sociology of pandemics, comparing their similarities and differences (Dingwall et al., 2013; Matthewman and Huppatz, 2020; Strong, 1990). This is a sociologically promising undertaking where, for example, the plague and the Spanish flu have left deep traces in the cultural stock of knowledge. Similarly, COVID-19 has been considered a ‘breaching experiment’ (Scambler, 2020) likely to trigger significant social changes from the ‘end of neoliberalism’ (Saad-Filho, 2020) to an end of ‘the world as we know it’ (Žižek, 2020). The COVID crisis is indeed exceptional in many respects, even when the impact amongst countries varied significantly from little to severe. However, the various once-in-a-lifetime crises of recent decades such as the Chernobyl disaster, the AIDS/HIV disease, the 9/11 attack, and indeed climate change, invite us to see the pandemic in a broader framework as of how risk, uncertainty and social change are experienced, approached and envisioned at different times and places.

For this purpose and informed by the sociology of risk and uncertainty, this concluding article focuses on three topics. Firstly, it traces learning opportunities, to be better prepared for future pandemics. Secondly, it examines some of the society–nature–technology assemblages and amalgamations which constituted the pandemic experience. Thirdly, it explores and envisions what all this means for the development of emerging subjectivities in response to the crisis.

## Opportunities for learning during the first wave

Since late 2019 experts in China and since early 2020 worldwide have tried to come to terms with the new virus, its dangers and how these could be controlled. From the beginning, this new and largely unknown threat challenged governments, experts and the public and they had to rely on a mix of uncertain knowledge and (professional) experience. However, alerted by rumours from China, Taiwan as other Asian countries responded early taking a proactive elimination strategy, looking back on negative experiences, for example with the 2003 SARS pandemic (Manch, 2020). In contrast, the Global North including the WHO responded more slowly and far less resolutely by emphasizing the need to be alert but by applying only a few far-reaching protective measures. Several reasons may explain this slow response, such as the lack of scientific evidence at the time and the still relatively low numbers of infections, the high economic costs for severe interruptions of social and economic life, and the large political costs should things turn out to be less severe than suspected. From a cultural symbolic perspective, there is another explanation for the West not following the precautionary approach. China in particular and Asia more generally have been considered for a long time as a distant Other, which has often been characterized as a source for infectious diseases because of lifestyles and living habits. Therefore, the policies of Western industrialized countries concentrated on containing diseases where they emerge and on isolating infected individuals if they should enter their country. In this way, routines had established through which the WHO typically channels support (technology, resources, etc.) to countries in need of development. Besides Asia, this equally applied to Africa and the Middle East where the international community helped to manage and control outbreaks such as SARS (2003), MERS (2012), and Ebola (2018–2020), which were successfully contained regionally within this established global division of labour. All this might have supported the feeling of being well prepared with plenty of time to respond and it nurtured the belief that COVID-19 would be managed within the highly developed health systems of the industrialized West. This attitude contrasted with the concerns in Taiwan, which had learned from the failures of managing the SARS pandemic in 2003 and the Chinese attempt to cover up the outbreak. There, a precautionary approach seemed beneficial based on a new pandemic response plan and the country’s institutional setup. Considering Taiwan’s and other countries’ successful approach to managing the coronavirus proactively, there is a clear opportunity for global learning that requires the West to overcome the established global division of labour, socio-cultural prejudice and distancing mechanisms (Othering) as well as feelings of technological superiority.

Socio-cultural patterns were also observable when scientific experts debated and provided evidence to inform political decision-making. A compelling example was the debate in the West about whether to recommend wearing facemasks in public, which can be interpreted as a negotiation of culturally mediated practices and values. Even though scientific evidence provided clear figures on the degree facemasks can protect people against infection, the respective health authorities’ shifting advice resulted mainly from a change of an individualized to a population health perspective. There is still little evidence that a facemask can protect an individual over a longer time, but it reduces the transmission in the community when everyone wears a facemask in public. Even though wearing facemasks is a common and widely accepted means in response to infectious diseases in many countries in Asia, it was seen critically in the Global North, sometimes interpreted as a cultural oddity, lacking scientific evidence for its efficiency (Fleming, 2020). It was therefore not recommended by the WHO by the end of March 2020^1^ (Howard, 2020) and neither by many other countries of the Global North.^2^ However, the recommendations shifted soon, with a change in perspective. For example, the US Centers for Disease Control and Prevention (CDC) recommended wearing ‘cloth face coverings’ in public on 3 April (Jingnan, 2020).

Nevertheless, science played a crucial role in providing authority and trustworthiness to political leaders managing the crisis (Balog-Way and McComas, 2020; Dohle et al., 2020). In contrast to politicians, who are notoriously at the lower end of surveys on trustworthiness, science and doctors are at the top (NIM, 2016). Consequently, politicians often invited medical experts when announcing their decisions on how best to respond to the virus in the media. This worked particularly well when accompanied by strong leadership such as in New Zealand (Wardman, 2020). However, scientific knowledge cannot replace responsibility for political decisions since science lacks the political compass and legitimacy. For example, the decision to try an elimination strategy or a flattening the curve approach requires balancing different risks and accepts a difficult to calculate number of deaths. Such decisions cannot be made solely based on science. Politicians were therefore criticized when seen offloading (Henley, 2020) or attempting to cover political decisions with science (Runciman, 2020). Thus, good science is necessary for good pandemic management but requires political leadership to allow legitimate outcomes (Wardman, 2020).

Political leadership does not only require good evidence and a clear direction. As the example of successful management of the crisis showed, it also requires engaging the public and securing compliance with far-reaching measures, which cannot be fully controlled but require everyone to engage voluntarily to some extent. For example, Jacinda Ardern the PM of New Zealand talked live on social media about the burden of the measures and the need and reasons to follow them. This was also accompanied by a successful educational campaign during which Dr Ashley Bloomfield, Director General of Health, not only engaged in one-way explanation of the situation and the measures but engaged in a dialogue on the (social) media answering people’s questions and concerns and addressing conspiracy theories and fake news (Roy, 2020). Engagement of the public in decision-making is one of the most effective ways of risk communication and crucial for generating legitimacy in democratic societies (Fischhoff, 1995). Even though the public is not engaged in the decision-making process, risk communication that opens discussions and provides justification reduces the social distance to and increases acceptance of politicians and the decisions they make. Making politicians ‘common’ also increased trust in the government’s risk management. When in New Zealand the Health Minister did not comply with quarantine rules he was publicly named and punished, showing that there are no exceptions (BBC, 2020). This contrasted sharply with the UK government’s backing of government advisor Dominic Cummings when breaching the rules (Fancourt et al., 2020) and was followed by drops in support and trust in the government’s pandemic response.

The relevance for the social dimension to secure engagement and compliance with the COVID response becomes also clear in cases where the pandemic was downplayed, as in the case of the US Trump government. Good evidence and scientific expertise played little importance in generating support for the position of negligence. This can be explained by the two-dimensional character of trust which combines a cognitive and knowledge-based dimension with a more emotional and social relationship-based dimension (Barbarlet, 2009). The social dimension was central for mobilizing social support for the contradictory responses to the crisis. Social media have played a significant role, not only because they have reshaped the public sphere in recent decades, but in supporting a historical trend of linguistically lowering social divisions as well as subjectification of language (Baker, 2017). Politicians and experts following this trend are perceived to be more directly and personally reachable and are more likely to foster trust and compliance.

There had been many ways in which global social differences shaped the responses to the pandemic. In the early days, in many democratic societies in Europe privacy concerns and the restrictions of civil rights were a key topic in public debate. In contrast to China and other Asian countries, many governments of the Global North were reluctant to restrict people’s movements efficiently at the beginning of the pandemic. Automated surveillance with the help of digital technologies to trace contacts or even monitor compliance was rejected as a threat to democracy and individual freedom thereby restricting the use of technological tools for the management of the pandemic which had been successfully applied elsewhere (Dudden and Marks, 2020). Although the use of automated contact tracing tools was considered, it had to be balanced against privacy concerns. As a consequence, a contact tracing app that had been developed to meet privacy needs in Germany was highly inefficient and played hardly any role in managing the pandemic (DW, 2020) in contrast to the apps which swiftly had become part of successful pandemic management regimes in Singapore, Taiwan and South Korea. This raises questions about the possibility of the development of technologically better suitable apps as well as the costs and difficulties to conduct efficient contact tracing on a large scale without efficient technical support. New solutions might be possible which serve both data protection and privacy norms as well as pandemic management needs.

When socio-cultural values were affected, governments followed different approaches. China cancelled the Lunar New Year celebrations over deadly virus fears and put several mega-cities under lockdown in January 2020 (DW, 2020), and Iran started to cancel public events from February onwards but delayed the closure of the Shia shrines in Qom until mid-March (Kursun, 2020). Similarly, many governments in Europe were prepared to weaken necessary measures of efficient pandemic management over religious issues. For example, during Christmas, Germany eased restrictions for face-to-face meetings permitting relatives to celebrate together, which resulted in a significant increase of infections and a setback in pandemic management in early 2021. India allowed a huge Hindu festival in the Ganges river amid the second wave of the pandemic in 2021 turning it into a super-spreader event with the subsequent overburdening of the health system (DW, 2021). Such cases raise important questions about the tension of socio-cultural pressures to maintain deeply socially rooted rituals to potentially high costs and whether there are better ways of balancing them.

Many problems occurred and were responded to in different ways, such as the increasing domestic violence under lockdown (Townsend, 2020). However, the protection of the vulnerable did not work out well considering that in many countries from 30 to more than 50% died in nursing and retirement homes (Booth, 2020b; Comas-Herrera et al., 2021) while marginalized groups more generally were highly affected for a mix of different reasons (e.g. living conditions, health, language). The responses often dominated by epidemiological considerations were not sensitive to the needs and vulnerabilities of people at the social margins. As a result, general socio-structural factors remained good predictors for the likelihood to become infected and to die with COVID-19 (Booth, 2020a; Evelyn, 2020).

In summary, several key issues challenged social responses to the pandemic. Socio-cultural, institutional as well as deeply rooted socio-structural aspects shaped the experience and responses to the crisis on a global and national level. The contributions to this monograph issue have touched already on several key elements shaping the social influence of the pandemic, such as social inequality, Othering and stigma, trust, everyday risk rationales, risk communication, public discourses, and global risk dynamics. As this monograph has highlighted, concrete practices and debates shaped by socio-cultural dimensions influenced the responses of politicians and the public likewise.

However, the pandemic threat has been experienced and has unfolded very differently on a global scale and consequently, governments provided varied responses to different problems. As a result, countries that have eliminated the virus are predominantly concerned with controlling new outbreaks. Those trying to ‘flatten the curve’ explore different behavioural policies, while in countries where infection rates went out of control the focus is put on stabilizing an overburdened health care system. The following explores how the combination or amalgamation of social, natural and technological aspects result in different pandemic ‘realities’.

## Pandemic assemblages of humans, nature and technology

The coronavirus crisis has highlighted the complex ways in which society, nature and technology combine encouraging greater openness to the complex relationships between humans and non-humans as has been emphasized by material semiotics (Latour, 1999; Law, 2008). COVID-19 is an excellent opportunity to examine how living beings, whether human or non-human, and non-human physical environments (including technology) establish complex networks. Looking back at the debates about the reality of the virus illustrates how this reality of the spreading virus differed and influenced perceptions over time. Most outstanding was the opportunity some countries took to profit from their geographic advantage in controlling their borders when successfully engaging in a virus elimination strategy. Further examples show social constructions responding to changing realities as well as social debate referring to different social realities such as in the case of facemask-wearing and the shift from individual protection to population protection. Moreover, the pandemic provided an opportunity to explore not only how humans and their natural environment relate but also how technology is involved. This includes the destruction of the environment, the provision of the means to fight the virus and – for people who have access – the digital environment that helped them to manage the crisis. The following outlines some examples for the different interconnections to illustrate these points.

The social existence of the virus started with doctors in Wuhan experiencing patients with symptoms that were similar to the 2003 SARS outbreak. Their concerns were not based on scientific evidence yet, it was based on doctors’ practical knowledge and was raised by a doctor working at the hospital (Buckley and Myers, 2020). With scientific evidence still lacking authorities pushed back the claims of a new coronavirus until they had isolated the virus (Buckley, 2020). However, knowledge was still patchy, even when human-to-human transmission might have been likely it required the official scientific confirmation to what extent it took place. The Chinese authorities might have tried to cover up the outbreak as some observers suggested. But there is also a more general problem. Every emerging risk requires experts and decision-makers to balance scaremongering and trivialization (Giddens, 2002). In an age of heightened responsibilization proven scientific evidence is a resource that helps to deflect blame in case of undesired developments. However, waiting for research to be conducted delays responses and therefore invites possible negative outcomes. Therefore, good risk management often utilizes soft forms of experience-based knowledge to apply a precautionary approach. This might rest on non-knowledge rather than knowledge, or what could be called ‘well-informed guesswork’. While the WHO and many countries waited for further evidence for giving recommendations and organizing their responses, Taiwan’s response already took precautionary measures. Grounded on the experience with the SARS 2003 crisis, when China covered up the outbreak, Taiwan did not wait for official confirmation or scientific evidence. Instead, at times of lacking knowledge, they started immediately with testing arrivals from China even before they had their first case (Sui, 2020). Thus, Taiwan’s successful response was not based on knowledge but non-knowledge (Gross, 2007) and it started at the time when many other experts in the Global North were still hoping for a flu-like or easy controllable virus (e.g. Boorman, 2020; Johnson, 2020; Schmidt, 2020). The assumption that there would be time to respond in the event the virus turned out to be more serious gave time to the virus to further spread across countries and within communities. Thus, in the early days of the crisis, it was not so much a problem of knowledge being interpreted differently but how the lack of knowledge about the virus was approached, proactively or hesitantly.

Besides the management of non-knowledge, there were also good examples of the social factors shaping the scientific production of knowledge, which raised questions about the compatibility of scientific knowledge and practical knowledge (cf. also Wynne, 1996). An early study examined the first infections in Germany in a company in Bavaria where participants of a workshop have been infected by a visitor from China, to examine whether asymptomatic transmission takes place. Not having been able to talk to the spreader of the virus the study relied on the judgements of the participants, who did not notice significant health issues of the Chinese guest. Not having talked to the actual infected person is a methodological flaw of the online pre-published study and it was therefore criticized as unscientific. However, more important than the row between competing research teams (Kupferschmidt, 2020; Rothe et al., 2020) is the question of what can count as ‘socially asymptomatic’. Scientific knowledge is not directly applicable but requires translation and interpretation into the realm of people’s everyday knowledge, which follows its own standards and criteria of illness. What are symptoms in a scientific rationale might not be recognizable and distinguishable from normal everyday life issues, and not worth further consideration. Symptoms cannot be decontextualized. This extends the question about the right knowledge about reality to the analysis of knowledges of different physical realities. A related argument could be made regarding the argument about facemask-wearing.

The debate about the efficiency of facemask-wearing highlighted that evidence-based recommendations were shaped by many factors. One was the availability of sufficient numbers of facemasks to provide for care workers. The other was the problem formulation, whether facemasks should protect the wearer of the mask or the community. When interpreted in a material semiotics perspective this debate was not merely about cultural differences or different interpretations of the same reality but different realities (Mol, 2003). As a result, for both cases supportive evidence was available. With the change from the protection of individuals to the protection of populations, other evidence was underpinning the policy.

The society–nature–technology nexus is also useful when approaching the reality of a virus originating and quickly spreading from hotspots or mega spreading events. Several known events that were efficient in spreading the virus demonstrate how quickly the right conditions such as density of bodies and activities in a dance bar or soccer stadium would arise. For example, the highly mobile people who met in Ischgl, a popular ski resort in Austria, significantly contributed to the spread of the virus in Europe, with hundreds of cases in six countries being traced back to the town (Hoffower, 2020). There were even indications that it was one particular bar in Ischgl from which the virus spread, and suggestions that a specific habit such as playing oral beer pong and sharing whistles allowed the virus to easily spread through the exchange of saliva (Hruby, 2020). Similarly, the soccer match of Valencia (Spain) against Atalanta (Italy) in Milan on 19 February 2020 was considered a super-spreader event, suggested to have significantly accelerated the pandemic in Italy as well as Spain (Robinson, 2020). These and many more events of this kind served to turn a local infection into a global pandemic thanks to our technologies allowing people to quickly take the virus to foreign places and to infect others on the way and back home. Thereby, COVID-19 also challenged culturally embedded practices such as greeting one another (kissing left and right, shaking hands, etc.), which are often applied without thinking but have now become problematic and under suspicion as possible sources of infection.

It is hardly possible to understand the experience, management and long-term effect of the crisis without taking into account the accelerating digitization and digital divisions. Where available social digitization and in particular social media played a key role in the experience and responses to the pandemic. Often portrayed as a source of misinformation, social media have played a critical role in managing the crisis during a lockdown, for example as a resource of valuable information, to stay in contact with friends and relatives, or to maintain studies to give meaning and purpose to life under lockdown. Kaufmann et al.’s study (2020) on disrupted urban lives of young adults in Vienna gave some indication of the ambivalent experiences and possible lasting changes of young people’s subjectivities. Having intensified the use of digital media under lockdown, they explored and invented new ways of using them (e.g. using video conferencing tools to watch the same programme together). What has been a survival strategy for themselves and their well-being, keeping their social relationships going when physical personal contacts were restricted or not possible, has become an important resource (skills and routines) integrated into their life ever since or ready to fall back on during a future crisis. Indeed, everyone was keen to meet friends and relatives in person and with the length of lockdown experiences invented their own distancing standards when meeting face-to-face. But there were also young adults who re-evaluated the social pressure they had been under before and enjoyed having more time for themselves. Thus, social media might support people developing new ways of integrating digital media into their life to push back undesired social demands. It remains unclear whether this leads to having more containment of friendships through socializing digitally, keeping stronger control about the depth and commitments of a friendship (Turkle, 2011). The rapidly growing digital space of available activities, from visiting museums or cities virtually, doing various sports activities with trainers elsewhere in the world, educating yourself and much more, produces a new realm of opportunities that people can flexibly integrate into their life. In this way, the notion of ‘digital health’ might change insofar as it means using the extended realm of the digital world or the digital environment healthily both in terms of mental and physical well-being.

The society–nature–technology nexus relates well to research that found a connection between the growing numbers of zoonotic diseases and the destruction of the natural environment, including the loss of biodiversity (Johnson et al., 2020). This also connects well to the One World, One Health approach (Van Helden et al., 2013), which states that with natural habitats being increasingly destroyed human contacts with wild animals carrying a variety of pathogens become more regular, increasing the opportunity for viruses crossing the species boundaries. Turning to the micro-level Van Helden and colleagues (2013: 500) suggested that ‘we are only beginning to realise the beneficial effects of bacteria or viruses – not just the role of our gut microflora for digestion and health, but also the microbial zoo on our skin and in our environment’. As Hinchliffe (2015) noted, the focus on the transmission of pathogens of the One World, One Health approach could be extended by a social perspective for a truly interdisciplinary approach to flourish.

Taking the nexus of society, nature and technology into account helps to understand the dynamics of the coronavirus crisis. This relates not only to the capacities of health care systems to manage the virus but also the ability of society to manage necessary responses such as lockdowns, contact tracing, and to enforce compliance. Digital media have become an extended social environment which cannot replace face-to-face life but can help for a limited time to stay informed, stay connected and provide emotional support. The interesting question is how and to what extent the pandemic experience will change the social world in the years to come. Will public understanding and response to infectious diseases change attitudes towards infectious diseases more generally and possibly everyday life routines as well? How might indirect effects such as the acceleration of social digitization produce lasting changes in the social realm?

## Towards a new normal?

At times when most people are desperate to get their ‘normal’ life back, thinking about a ‘new normal’ after the coronavirus might be futile. Moreover, it is still unclear to what extent the vaccination campaigns of different countries will be sufficient to eliminate the virus, while some countries such as India are in the middle of a disastrous outbreak without enough vaccines, intensive care beds or oxygen available (Melimopoulos and Siddiqui, 2021). It is not even clear whether the virus will stay for some time even when most people are vaccinated, requiring the maintenance of routines of handwashing, social distancing, contact tracing and lockdowns. Neither is COVID-19 well enough known to predict its future behaviour nor can we know which mutations might develop in hotspots elsewhere in the world (Heise, 2021). Independently of how the current pandemic develops, when observations are correct that zoonotic diseases are likely to occur more often because of the ongoing destruction of the natural environment, it is desirable to take the social aspects into account when developing the technological and political tools for legitimate responses. Therefore, a better understanding of the new subjectivities, which shape public awareness, understanding, and likely responses, would be helpful for future pandemics since these can vary a lot depending on people’s concrete affectedness (e.g. death of relatives, own illness), milieu-specific living conditions (resources, lifestyles) and transnational divisions. Even though all generalizations remain problematic, some reflections might still be useful while being sensitive for their limited applicability.

One of the most pressing questions for future pandemics is how the experience of COVID-19 has changed people’s general understanding of viruses, epidemics and necessary responses. The likelihood to comply with curfews and self-isolation measures will still depend on other social conditions one lives under, such as the need to work for securing basic needs. But what about essential practices such as handwashing or the wearing of facemasks? From all that we know, behavioural change is most likely when supported by concrete individual experiences in one’s own life (Davis and Lohm, 2020). Having been severely ill with COVID-19 or having infected a friend or relative might lead to strong emotionally loaded memories, which inform future behaviour in contrast to having heard about the pandemic mainly from a distance through the news.

After facemask-wearing has been proven to be an efficient means to slow down the spread of the virus, it would be desirable to integrate facemask-wearing into the general behavioural repertoire in the global West, where prejudice and doubts have been strongest about their effectiveness while people still felt uncomfortable wearing them. Personal experience and own vulnerability might support a shift of attitude and behaviour in particular when facing a deadly pandemic again. It is less clear whether a more general shift will take place in the short-term that integrates facemask-wearing in the cultural repertoire as responsible behaviour to protect others. For example, in Japan in the early 20th century facemask-wearing became a symbol for the successful national effort to conquer the Spanish flu. It expressed the successful shift to modernity and became further normalized in different socio-historical contexts later. Wearing a facemask became a norm for every sick person to protect others, an attitude which was further encouraged with a growing individualism that emphasized the individual responsibility to follow the ‘cough etiquette’ to protect others (Horii, 2014). It is not yet clear whether facemask-wearing in the industrialized societies of the West will find such additional normative support to inform their routine usage, for example, to protect vulnerable others during the annual flu season.

The COVID-19 experience pushed at least partly the possibility for many employees to work from home if necessary, under the conditions that high-speed internet is available. Since this shift to working from a home office is only possible for specific occupations, the divide between work with spatial flexibility and work which requires physical presence is likely to widen with the additional division between labour with and without virus exposure. This comes with the double burden of risk of infection and risks resulting from the pandemic responses such as losing income when working in the informal labour market. The digital divide is also likely to grow in education. In many rich countries of the Global North educational institutions such as schools and universities were able to shift to online teaching and to build capacity to maintain online learning, which disadvantaged those without sufficient working conditions at home. However, in other countries, educational institutions were closed altogether for a lengthy period with yet unknown long-term effects.

The digital divide is likely not only to widen regarding work and education. Having access to digital media is a valuable resource to continue social life under pandemic conditions. Even when not supplanting face-to-face contacts it has been proven to be a crucial resource to keep informed, organize support, and continue social relationships (Kaufmann et al., 2020). The push for this generation to organize their life around digital media might have a double effect. Being more aware of the value of physical activities with friends as well as being more proficient in using and organizing social relationships with the help of digital media. Such skills might become crucial when work and educational conditions require more time flexibility (Woodman, 2012). Still, as the study of Kaufmann et al. (2020) showed, online activities remain overwhelmingly complementary rather than fully supplanting relationships to physical others. Where meeting others in the dense schedule of present-day social realities becomes more difficult, it might at the same time become more valued, planned for and celebrated when possible.

Considering living in a climate change world, the new experiences with virtual mobility has put pressure on long and short distance flights as one of the unnecessary sources of greenhouse gas production. With air travel likely to become more expensive and heightened cost pressure, companies have already indicated to realize savings through replacing physical travel with online meetings, even when face-to-face contacts remain crucial and prioritized in many work contexts. Other cost reductions could result from allowing more of the workforce to work from home.

It remains to be seen whether the experience of nature as a source of pathogens might contribute to changing general attitudes towards the natural environment from requiring protection and a benign and healthy place for recreation, to a place which comes with unknown risks requiring constant caution. Exercising caution towards unknown and hidden human-made dangers waiting to be discovered (e.g. contaminated sites) is then accompanied by the indirect hazards of the social and natural environment likely to change long-term attitudes and behaviour.

For the rich and ageing countries of the Global North COVID-19 was a wake-up call that they are vulnerable to viruses that were usually contained elsewhere. Nature and the viruses of wild animals not only come closer, but pandemic risks are also adding to other global risks requiring international collaboration. In Ulrich Beck’s (2009) perspective the cosmopolitan moment of the pandemic allows opportunities for cosmopolitanism to advance. To what extend the opportunity for global learning is taken up remains to be seen. There is little doubt, however, that pandemics will contribute to long-term changes in human attitudes and behaviour towards the environment and the technologically shaped lifeworld.

## Notes

1. There were also concerns that the recommendation to wear a facemask in public could cause further shortages of medical facemasks for frontline workers. But this does not concern self-made masks.

2. An exception was Slovakia (Šteňo, 2020).

## References

[bibr1-00113921211023518] Baker P (2017) American and British English. Divided by a Common Language? Cambridge: Cambridge University Press.

[bibr2-00113921211023518] Balog-Way DHP McComas KA (2020) COVID19: Reflections on trust, trade-offs, and preparedness. Journal of Risk Research 23(78): 838–848.

[bibr3-00113921211023518] Barbalet J (2009) A characterization of trust and its consequences. Theory and Society 38(4): 367–382.

[bibr4-00113921211023518] BBC (2020) Coronavirus: NZ health minister breaks lockdown at beach. 7 April. Available at: www.bbc.com/news/world-asia-52194407

[bibr5-00113921211023518] Beck U (2009) World at Risk. Cambridge: Polity.

[bibr6-00113921211023518] Boorman S (2020) Why our response to the corona virus should be: ‘Don’t Panic!’ Empactis, 28 January. Available at: https://empactis.com/why-our-response-to-the-corona-virus-should-be-dont-panic/

[bibr7-00113921211023518] Booth R (2020a) BAME groups hit harder by Covid-19 than white people, UK study suggests. The Guardian, 7 April. Available at: www.theguardian.com/world/2020/apr/07/bame-groups-hit-hardercovid-19-than-white-people-uk

[bibr8-00113921211023518] Booth R (2020b) Half of coronavirus deaths happen in care homes, data from EU suggests. The Guardian, 13 April. Available at: www.theguardian.com/world/2020/apr/13/half-of-coronavirusdeaths-happen-in-care-homes-data-from-eu-suggests

[bibr9-00113921211023518] Buckley C (2020) Chinese doctor, silenced after warning of outbreak, dies from coronavirus. The New York Times, 6 February. Available at: www.nytimes.com/2020/02/06/world/asia/chinese-doctor-Li-Wenliang-coronavirus.html (accessed 30 April 2020).

[bibr10-00113921211023518] Buckley C Myers SL (2020) A new coronavirus spread, China’s old habits delayed fight. New York Times, 1 February. Available at: www.nytimes.com/2020/02/01/world/asia/china-coronavirus.html (accessed 30 April 2020).

[bibr11-00113921211023518] Comas-Herrera A Zalakaín J Lemmon E , et al. (2021) Mortality associated with COVID-19 in care homes: International evidence. LTCcovid.org, International Long-Term Care Policy Network, CPEC-LSE, 1 February.

[bibr12-00113921211023518] Davis M Lohm D (2020) Pandemics, Publics, and Narrative. Oxford: Oxford University Press.

[bibr13-00113921211023518] Dingwall R Hoffman LM Staniland K (2013) Introduction: Why a sociology of pandemics? Sociology of Health and Illness 35(2): 167–173. 23278292 10.1111/1467-9566.12019

[bibr14-00113921211023518] Dohle S Wingen T Schreiber M (2020) Acceptance and adoption of protective measures during the COVID19 pandemic: The role of trust in politics and trust in science. Social Psychological Bulletin. OSF Preprints, 29 May. doi:10.31219/osf.io/w52nv

[bibr15-00113921211023518] Dudden A Marks A (2020) South Korea took rapid, intrusive measures against Covid-19 – and they worked. The Guardian, 20 March. Available at: www.theguardian.com/commentisfree/2020/mar/20/south-korea-rapid-intrusive-measures-covid-19

[bibr16-00113921211023518] DW (2020) China cancels Lunar New Year events over deadly virus fears. DW, 23 January. Available at: www.dw.com/en/china-cancels-lunar-new-year-events-over-deadly-virus-fears/a-52121516

[bibr17-00113921211023518] DW (2021) India: Hindu festival turns to superspreader event as COVID infections soar. DW, 15 April. Available at: www.dw.com/en/india-hindu-festival-turns-to-superspreader-event-as-covid-infections-soar/a-57218497

[bibr18-00113921211023518] Evelyn K (2020) ‘It’s a racial justice issue’: Black Americans are dying in greater numbers from Covid-19. The Guardian, 8 April. Available at: www.theguardian.com/world/2020/apr/08/its-a-racial-justiceissue-black-americans-are-dying-in-greater-numbers-from-covid-19 (accessed 14 April 2020)

[bibr19-00113921211023518] Fancourt D Steptoe A Wright L (2020) The Cummings effect: Politics, trust, and behaviours during the COVID-19 pandemic. The Lancet 396(10249): P464–465. 10.1016/S0140-6736(20)31690-1 PMC7613216 32771083

[bibr20-00113921211023518] Fischhoff B (1995) Risk perception and communicating unplugged: Twenty years of process. Risk Analysis 15: 137–145. 7597253 10.1111/j.1539-6924.1995.tb00308.x

[bibr21-00113921211023518] Fleming A (2020) Our face mask future: Do they really help beat flu, coronavirus and pollution? The Guardian, 31 January. Available at: www.theguardian.com/world/2020/jan/31/our-face-mask-future-do-they-really-help-beat-flu-coronavirus-and-pollution

[bibr22-00113921211023518] Giddens A (2002) Runaway World: How Globalisation is Reshaping our Lives. London: Profile Books.

[bibr23-00113921211023518] Gross M (2007) The unknown in process: Dynamic connections of ignorance, non-knowledge and related concepts. Current Sociology 55(5): 742–759.

[bibr24-00113921211023518] Heise G (2021) The COVID variant from India: What we know so far. DW, 27 April. Available at: www.dw.com/en/the-covid-variant-from-india-what-we-know-so-far/a-57313664

[bibr25-00113921211023518] Henley J (2020) Swedish PM warned over ‘Russian roulette-style’ Covid-19 strategy. The Guardian, 23 March. Available at: www.theguardian.com/world/2020/mar/23/swedish-pm-warned-russian-roulette-covid-19-strategy-herd-immunity (accessed 20 May 2020).

[bibr26-00113921211023518] Hinchliffe S (2015) More than one world, more than one health: Re-configuring interspecies health. Social Science & Medicine 129: 28–35. 25022470 10.1016/j.socscimed.2014.07.007

[bibr27-00113921211023518] Hoffower H (2020) Inside ‘ground zero’ of Europe’s coronavirus pandemic, the Austrian ski resort under investigation for spreading the coronavirus across 6 countries and allegedly trying to cover it up. Business Insider, 24 April. Available at: www.businessinsider.com/ischgl-ski-resort-coronavirus-pandemic-lawsuit-investigation-2020-4?r=DE&IR=T

[bibr28-00113921211023518] Horii M (2014) Why do the Japanese wear masks? Electronic Journal of Contemporary Japanese Studies 14(2): art. 8.

[bibr29-00113921211023518] Howard J (2020) To help stop coronavirus, everyone should be wearing face masks. The science is clear. The Guardian, 4 April. Available at: www.theguardian.com/commentisfree/2020/apr/04/why-wear-a-mask-may-be-our-best-weapon-to-stop-coronavirus

[bibr30-00113921211023518] Hruby D (2020) How an Austrian ski resort helped coronavirus spread across Europe. CNN, 24 March. Available at: https://edition.cnn.com/2020/03/24/europe/austria-ski-resort-ischgl-coronavirus-intl/index.html

[bibr31-00113921211023518] Jingnan H (2020) Why there are so many different guidelines for face masks for the public. NPR, 10 April. Available at: www.npr.org/sections/goatsandsoda/2020/04/10/829890635/why-there-so-many-different-guidelines-for-face-masks-for-the-public (accessed 13 April 2020).

[bibr32-00113921211023518] Johnson CK Hitchens PL Pandit P , et al. (2020) Global shifts in mammalian population trends reveal key predictors of virus spillover risk. Proceedings of the Royal Society B, Biological Science. https://royalsocietypublishing.org/doi/pdf/10.1098/rspb.2019.2736 10.1098/rspb.2019.2736 PMC7209068 32259475

[bibr33-00113921211023518] Johnson CY (2020) Scientists are unraveling the Chinese coronavirus with unprecedented speed and openness. The Washington Post, 24 January. Available at: www.washingtonpost.com/science/2020/01/24/scientists-are-unraveling-chinese-coronavirus-with-unprecedented-speed-openness/

[bibr34-00113921211023518] Kaufmann K Straganz C Bork-Hüffner T (2020) City-life no more? Young adults’ disrupted urban experiences and their digital mediation under Covid-19. Urban Planning 5(4): 324–334.

[bibr35-00113921211023518] Kupferschmidt K (2020) Study claiming new coronavirus can be transmitted by people without symptoms was flawed. AAAS, 3 February. Available at: www.sciencemag.org/news/2020/02/paper-non-symptomatic-patient-transmitting-coronavirus-wrong (accessed 13 May 2020).

[bibr36-00113921211023518] Kursun M (2020) COVID-19: Iran’s holiest sites closing for visitors. Anadolu Agency, 16 March. Available at: www.aa.com.tr/en/health/covid-19-irans-holiest-sites-closing-to-visitors/1768192

[bibr37-00113921211023518] Latour B (1999) Reassembling the Social: An Introduction to Actor-Network-Theory. Oxford: Oxford University Press.

[bibr38-00113921211023518] Law J (2008) Actor-network theory and material semiotics. In: Turner BS (ed.) The New Blackwell Companion to Social Theory, 3rd edn. Oxford: Blackwell, pp. 141–158.

[bibr39-00113921211023518] Manch T (2020) Coronavirus: New Zealand likely to follow Taiwan in advising cancellation of crowded gatherings. Stuff, 15 March. Available at: www.stuff.co.nz/national/health/coronavirus/120294477/coronavirus-new-zealand-likely-to-follow-taiwan-in-advising-cancellation-of-crowded-gatherings (accessed 15 May 2020).

[bibr40-00113921211023518] Matthewman S Huppatz K (2020) A sociology of Covid-19. Journal of Sociology 56(4): 675–683.

[bibr41-00113921211023518] Melimopoulos E Siddiqui U (2021) WHO says rush to hospitals worsens India COVID crisis. Aljazeera, 27 April. Available at: www.aljazeera.com/news/2021/4/27/india-receives-first-shipment-of-uk-covid-medical-aid-live-news

[bibr42-00113921211023518] Mol A (2003) The Body Multiple. Durham, NC: Duke University Press.

[bibr43-00113921211023518] NIM (Nuremberg Institute for Market Decisions) (2016) Worldwide ranking: Trust in professions. March. Available at: www.nim.org/en/compact/focustopics/worldwide-ranking-trust-professions

[bibr44-00113921211023518] Robinson J (2020) The soccer match that kicked off Italy’s coronavirus disaster: Decision to hold Atlanta-Valencia Champions League match in February accelerated spread of pandemic. The Wall Street Journal, 1 April. Available at: www.wsj.com/articles/the-soccer-match-that-kicked-off-italys-coronavirus-disaster-11585752012

[bibr45-00113921211023518] Rothe C Schunk M Sothmann P , et al. (2020) Transmission of 2019-nCoV infection from an asymptomatic contact in Germany. The New England Journal of Medicine 382(10): 970–971. 32003551 10.1056/NEJMc2001468 PMC7120970

[bibr46-00113921211023518] Roy EA (2020) ‘Delivers the stats like no other’: New Zealand’s Covid-19 crush on health chief. The Guardian, 10 April. Available at: www.theguardian.com/world/2020/apr/10/delivers-the-stats-like-no-other-new-zealands-covid-19-crush-on-health-chief

[bibr47-00113921211023518] Runciman D (2020) Coronavirus has not suspended politics – it has revealed the nature of power. The Guardian, 27 March. Available at: www.theguardian.com/commentisfree/2020/mar/27/coronavirus-politics-lockdown-hobbes

[bibr48-00113921211023518] Saad-Filho A (2020) From COVID-19 to the end of neoliberalism. Critical Sociology 46(4–5): 477–485. 10.1177/0896920520929966 PMC7262736 38602967

[bibr49-00113921211023518] Scambler G (2020) Covid-19 as a ‘breaching experiment’: Exposing the fractured society. Health Sociology Review 29(2): 140–148. 33411650 10.1080/14461242.2020.1784019

[bibr50-00113921211023518] Schmidt F (2020) Opinion: Coronavirus paranoia is outpacing its actual danger. DW, 28 January. Available at: www.dw.com/en/opinion-coronavirus-paranoia-is-outpacing-its-actual-danger/a-52179519

[bibr51-00113921211023518] Šteňo A (2020) Facemasks against COVID-19: Why Slovakia became the trailblazer. EUROCTIV, 28 April. Available at: www.euractiv.com/section/coronavirus/opinion/facemasks-against-covid-19-why-slovakia-became-the-trailblazer/

[bibr52-00113921211023518] Strong PM (1990) Epidemic psychology: A model. Sociology of Health and Illness 12(3): 249–259.

[bibr53-00113921211023518] Sui C (2020) What Taiwan can teach the world on fighting the coronavirus. NBC, 10 March. Available at: www.nbcnews.com/health/health-news/what-taiwan-can-teach-world-fighting-coronavirus-n1153826

[bibr54-00113921211023518] Townsend M (2020) Revealed: Surge in domestic violence during Covid-19 crisis. The Guardian, 12 April. Available at: www.theguardian.com/society/2020/apr/12/domestic-violence-surges-seven-hundred-per-cent-uk-coronavirus

[bibr55-00113921211023518] Turkle S (2011) Alone Together: Why We Expect More from Technology and Less from Each Other. New York: Basic Books.

[bibr56-00113921211023518] Van Helden PD Van Helden LS Hoal EG (2013) One world, one health. EMBO Reports 14(6): 497–501. 23681441 10.1038/embor.2013.61 PMC3674448

[bibr57-00113921211023518] Wardman JK (2020) Recalibrating pandemic risk leadership: Thirteen crisis ready strategies for COVID-19. Journal of Risk Research 23(7–8): 1092–1120.

[bibr58-00113921211023518] Woodman D (2012) Life out of synch: How new patterns of further education and the rise of precarious employment are reshaping young people’s relationships. Sociology 46(6): 1074–1090.

[bibr59-00113921211023518] Wynne B (1996) Misunderstood misunderstandings: Social identities and public uptake of science. In: Irwin A Wynne B (eds) Misunderstanding Science? The Public Reconstruction of Science and Technology. Cambridge: Cambridge University Press, pp. 19–46.

[bibr60-00113921211023518] Žižek S (2020) Pandemic! Covid-19 Shakes the World. New York: OR Books.

